# An Update on Surgical versus Expectant Management of Ovarian Endometriomas in Infertile Women

**DOI:** 10.1155/2015/204792

**Published:** 2015-07-09

**Authors:** Sanaz Keyhan, Claude Hughes, Thomas Price, Suheil Muasher

**Affiliations:** ^1^Division of Reproductive Endocrinology and Infertility, Duke University Medical Center, 5704 Fayetteville Road, Durham, NC 27713, USA; ^2^Cardiovascular & Metabolic Diseases, Medical Strategy & Science, Quintiles, Inc., 5927 South Miami Boulevard, Morrisville, NC 27560, USA; ^3^Department of Obstetrics and Gynecology, Duke University Medical Center, Durham, NC 27710, USA

## Abstract

Ovarian endometriomas are a common manifestation of endometriosis that can represent a more severe stage of the disease. There is much debate over the treatment of these cysts in infertile women, particularly before use of assisted reproductive technologies. Evidence exists that supports surgical excision of ovarian endometriomas, as well as evidence that cautions against surgical intervention. Certain factors need to be examined closely before proceeding with surgery or continuing with expectant management. These include the patient's symptoms, age, ovarian reserve, size and laterality of the cyst, prior surgical treatment, and level of suspicion for malignancy. The most recent evidence appears to suggest that certain patient profiles may benefit from proceeding directly to in vitro fertilization (IVF). These include symptomatic infertile patients, especially those that are older, those that have diminished ovarian reserve, those that have bilateral endometriomas, or those that have had prior surgical treatment. Although endometriomas can be detrimental to the ovarian reserve, surgical therapy may further lower a woman's ovarian reserve. Nevertheless, the presence of an endometrioma does not appear to adversely affect IVF outcomes, and surgical excision of endometriomas does not appear to improve IVF outcomes. Regardless of treatment plan, infertile patients with endometriomas must be counseled appropriately before choosing either treatment path.

## 1. Introduction

Endometriosis is a common gynecological problem affecting 6–10% of women of reproductive age. These women may be asymptomatic, but the majority will present with pelvic pain, infertility, or an adnexal mass. In fact endometriosis has been reported to be as high as 35–50% in women presenting with infertility [1]. The true prevalence of endometriosis in infertile women is difficult to ascertain, ranging from 9 to 50% in the literature, partly due to the requirement of a surgical diagnosis. In 2012, the Practice Committee of the American Society for Reproductive Medicine no longer recommended performing laparoscopy on asymptomatic women with infertility to check for endometriosis, making it even more difficult to quantify the true prevalence [2].

An endometrioma is the formation of a cyst within the ovary with ectopic endometrial tissue lining. This is one of the most common manifestations of endometriosis. Endometriomas are found in 17–44% of patients with endometriosis [3]. The prevalence of endometriomas is much easier to determine since the diagnosis is based on ultrasound. The sensitivity and specificity of diagnosis via ultrasound are 73% and 94%, respectively [4]. Color Doppler can help to identify vascularization of the mass and some authors have found that ovarian endometriomas in women with pelvic pain are more vascularized than in asymptomatic women [5]. Three-dimensional ultrasound provides new and unique ways of assessing ovarian cysts and is becoming more popular in clinical practice. Alcázar et al. reported that B-mode ultrasound with the use of mean gray value has a sensitivity of 80% and specificity of 91% in discriminating endometriomas from other unilocular cysts in premenopausal women [6]. Another imaging modality for examining endometriomas is magnetic resonance imaging (MRI). Endometriomas usually present as hyperintense signals on fat-suppressed T1-weighted imaging with a sensitivity of 90%, specificity of 98%, and accuracy of 96% [7]. Advanced techniques in MRI can further assist in the differentiation of endometriomas from other ovarian cysts, including hemorrhagic functional cysts.

There are three theories for formation of endometriomas. The first was described by Hughesdon in 1957 in which he suggested that there is an invagination of the ovarian cortex after accumulation of menstrual debris from bleeding of endometrial implants which results in a pseudocyst [8]. In 1994 Brosens et al. demonstrated through ovarioscopy that in most cases endometriomas are formed by invagination of the cortex and that active implants are located at the site of invagination [9]. The second theory is that endometriomas result from metaplasia of coelomic epithelium covering the ovary [10, 11]. Finally, Nezhat et al. have postulated that large endometriomas may develop as a result of secondary involvement of functional ovarian cysts by endometrial implants located on the ovarian surface [12].

Although an ovarian endometrioma is described as an ovarian cyst, its pathology is rather complex and completely different from other benign ovarian cysts. The majority of endometriomas are thought to be pseudocysts as described by Hughesdon rather than intraovarian cysts [8, 13]. The plane of cleavage between an endometrioma and ovarian cortex may not always exist like in other benign ovarian cysts. In a retrospective study, Scurry et al. examined two oophorectomy and 27 cystectomy specimens from women with endometriomas under the age of 35 years. These authors wanted to determine whether histological examination is of use in the classification of endometriotic cysts based on the three theories of endometrioma pathogenesis and if so whether classification is of clinical relevance. They placed endometriotic cysts into four different categories: (i) cortical invagination cysts; (ii) surface inclusion cyst-related endometriotic cysts; (iii) physiological cyst-related endometriotic cysts; and (iv) unclassified type. They found that ovarian cystectomy specimens were more difficult to categorize as the surgical excision of these cysts leads to adherence between the cyst and underlying ovarian parenchyma, artificial planes of cleavage, and fragmentation and tearing of the tissue during excision. The most common diagnosable cysts were the cortical invagination cysts. The majority of cysts, however, were the unclassifiable type either because the endometriosis process had destroyed evidence of their pathogenesis or because they are derived from intraparenchymal endometriotic deposits of undetermined origin [14].

Endometriomas are associated with a more severe form of the disease and do not respond well to medical therapy. This therapy may be able to improve pain or reduce the size of the cyst but it will not improve infertility [10, 15]. Therefore, the focus has been on surgical treatment in an attempt to improve fertility.

The exact mechanism by which endometriomas cause infertility is not known. Many authors have shown that there is a decrease in ovarian reserve and follicular density in women with endometriomas possibly due to an increase in oxidative stress [16]. Paradoxically, surgical resection of these cysts has been shown to further decrease ovarian reserve [15]. These findings have led to a great deal of controversy regarding the treatment of endometriomas in the setting of infertility, particularly in women who are undergoing assisted reproductive technology (ART). ART procedures not only create an economic burden, but also emotional and psychological stress. It is therefore imperative to elucidate the effect of ovarian endometriomas on fertility and ART procedures and, more importantly, the role of surgery.

In this paper, we will review the most recent literature regarding the impact of endometriomas on ovarian reserve and the pros and cons of surgical management.

## 2. Materials and Methods

This literature review was conducted by using the PubMed database of English literature (search terms: endometrioma AND infertility, surgery, ovarian reserve, assisted reproductive technologies) from 2008 to 2014 and cross-referencing. Meta-analyses, literature reviews, randomized controlled trials, and cohort studies were given priority. Our literature search produced arguments both in favor of and against surgical management of endometriomas.

## 3. Results/Discussion

### 3.1. Arguments for Surgical Excision

Evidence indicates that the primary benefit of surgical treatment of endometriosis is relief of pelvic pain. A Cochrane review in 2008, including two randomized controlled trials, concluded that laparoscopic excision of an endometrioma is associated with a decrease in symptoms of dysmenorrhea, dyspareunia, and nonmenstrual pelvic pain [17].

The presumptive benefit of surgical treatment to reduce or reverse the inherently damaging effects of endometriomas on the ovarian cortex is more controversial. There may be presurgical endometriosis-mediated damage to ovarian reserve beyond the stretching of ovarian cortex that can lead to loss of primordial follicles [16, 18]. Several studies have shown that there is a loss of follicular density in ovaries with endometriomas compared with unaffected ovaries [18–20]. One of the theories behind this follicular loss is that there is increased oxidative stress in the ovarian cortex surrounding endometriomas [16, 21, 22]. Sanchez et al. showed that there are not only increased reactive oxygen species inside the cyst, but also free iron which can be taken up by cells that are in direct contact. This may cause a gonadotoxic insult to individual follicles developing adjacent to the cyst. The authors of this study did acknowledge that their observations did not help to clarify if the surgical removal of the cyst or the presence of the cyst itself is more damaging to the otherwise healthy tissue [16, 21]. In another recent study, Kitajima et al. elucidated a different mechanism of follicular loss. They introduced the “burnout” hypothesis which states the following: endometriomas cause focal inflammation in the ovarian cortex leading to fibrosis and loss of cortex-specific stroma. This inflammation, with associated reduced vascularization and increased oxidative stress, may then lead to enhanced follicular recruitment and atresia resulting in a decline in the antral follicle count (AFC) [23]. Qiu et al. have demonstrated this reduced vascularization by detecting ovarian interstitial blood flow changes and increased blood flow resistance indices in women with endometriomas by using transvaginal color Doppler sonography. They suggest that these changes are indicative of ovarian interstitial fibrosis and microvascular injury. These authors recommend the use of transvaginal color Doppler sonography as a method of monitoring an ovarian endometrioma cyst-induced injury to surrounding ovarian interstitial vessels [24].

In several recent articles, Brosens et al. have emphasized that the management of an endometrioma must focus on the complex pathology of this condition as described above. They have confirmed through ovarioscopy-guided biopsies that endometriomas in situ show progressive smooth muscle cell metaplasia and fibrosis of the cortical layer. Additionally, they have found no correlation between the size of the endometrioma and the degree of ovarian pathology. They recommend that due to this inflammatory process ectopic endometrial tissue should be removed sooner rather than later irrespective of the cyst size. They describe a technique using transvaginal hydrolaparoscopy to not only diagnose an endometrioma but also ablate the cyst at an early stage in a minimally invasive fashion. Through transvaginal ovarioscopy or endoscopy of the endometriotic cyst, the endoscopic surgeon can evaluate the macroscopic pathology and determine the most appropriate surgical procedure, which may be through transvaginal surgery [13, 25].

Such studies have clearly shown that an endometrioma per se can have a gonadotoxic effect on the surrounding follicles. However, only a few studies have looked at the functional consequences and clinical implications of this insult. In one small observational study, Somigliana et al. showed reduced responsiveness to ovarian stimulation in women with unilateral endometriomas [26]. They found a decrease in the number of codominant follicles developing in the affected ovary compared with the contralateral unaffected ovary of the same patient. In another study, Benaglia et al. found that endometriomas have a detrimental impact on ovarian physiology by affecting ovulation [27]. The rate of ovulation in ovaries with endometriomas was noted to be significantly less than that of unaffected healthy ovaries. Barri et al. went one step further and studied pregnancy rates in women with endometriomas and found that expectant management of infertile women with endometriomas was associated with a pregnancy rate of 12% versus a conception rate of 54.2% in women who had surgical removal of their cysts [28].

Another persuasive argument favoring surgical excision of endometriomas relates to the dangers of expectant management such as ovarian torsion, cyst rupture, progression of endometriosis, or the threat of ovarian malignancy [15]. The two largest series reported a risk of occult malignancy in endometriotic cysts at frequencies of 0.8% and 0.9% [29, 30]. In a pooled analysis of 13 ovarian cancer case-control studies, endometriosis was associated with a significantly increased risk of invasive low grade serous, clear cell, and endometrioid ovarian cancers [31]. This study was limited by the fact that the presence of endometriosis was self-reported. Nevertheless, there appears to be a rare but calculable association between endometriosis and ovarian cancer.

Finally, removal of endometriomas may allow for better access of follicles at the time of oocyte retrieval. This technical utility is in addition to the benefit of preventing some pelvic infections after inadvertent drainage of the cyst [32–35]. Benaglia et al. reported that 2.8% of patients had accidental puncture of the endometrioma during oocyte retrieval, but no infections occurred as a result [36]. Regardless, according to the 2014 European Human Reproduction and Embryology (ESHRE) guidelines for management of women with endometriosis, antibiotics should be given prior to transvaginal oocyte retrieval in patients with endometriomas [37].

### 3.2. Arguments against Surgical Excision

Given that the presence of an endometrioma can be detrimental to the surrounding ovarian tissue, it is extremely important to be cognizant of a woman's ovarian reserve before proceeding to surgery. Several studies have shown this preoperative reduction in ovarian reserve by analyzing the commonly used biomarker of ovarian reserve, anti-Mullerian hormone (AMH) levels [38–40]. In a retrospective case-control study, Kim et al. found that preoperative AMH levels were significantly lower in women with Stage IV endometriosis who had endometriomas, compared to age-matched controls. This difference was not observed in women with Stage III endometriosis [38]. AMH was not affected by the size of the endometrioma or laterality. This is in contrast to a retrospective study by Hwu et al. that found women with bilateral endometriomas had significantly lower AMH levels compared to those with unilateral disease [39]. In both of these studies, the authors recommend checking preoperative AMH before performing an ovarian cystectomy and to use this information when counseling women before surgery, especially in those with low baseline AMH.

Furthermore, many publications including two recent meta-analyses have raised concern over the deleterious effects of ovarian cystectomy on ovarian reserve, specifically as reflected by AMH levels [41, 42]. One small study of 13 women with endometriomas showed that AMH can recover 3 months postoperatively; however most investigators have shown a decrease in AMH that can be sustained for up to 6–9 months even in the hands of experienced laparoscopic surgeons [3, 40–45]. In a prospective study of 30 women with endometriomas and 30 age-matched controls, Uncu et al. demonstrated that surgical excision of endometriomas leads to a decline in AMH that appears progressive [40]. Although the reduction in serum AMH was more prominent in women with excision of bilateral endometriomas versus unilateral endometriomas, the difference between these two groups was not statistically significant. The authors recognize that this difference may not have been evident due to the small sample size.  More recently, Alborzi et al. reported a significant decline in AMH up to 9 months after laparoscopic cystectomy in 193 women with endometriomas [3]. Unlike Uncu et al., these authors found a more significant decline of AMH in women with bilateral endometriomas, which is in agreement with Celik et al. and Hirokawa et al. [45, 46]. In fact, in a prospective study of 68 women with endometriomas, Kwon et al. reported that bilaterality was the only statistically significant factor in postoperative AMH decline [47]. There does also appear to be an association between the cyst size and postoperative AMH regression [3, 40, 46, 47]. Celik et al. found that there was a greater decline in AMH if the removed endometrioma was ≥5 cm [45].

The research regarding the effect of laparoscopic cystectomy of an endometrioma on the antral follicle count has been conflicting. Some argue that AFC may better reflect the specific damage to the operated ovary since this biomarker controls for the laterality of the injury. AMH, on the other hand, reflects the ovarian reserve of both gonads and can be influenced by compensation of the healthy ovary for the reduced reserve of the affected ovary [48].

Celik et al. reported an increase in AFC 6 months postoperatively despite a decrease in AMH [45]. The authors speculated that AFC cannot be a reliable ovarian reserve marker after endometrioma excision. This is because the presence of an endometrioma may underestimate the measurement of AFC preoperatively. Alborzi et al. and Biacchiardi et al. also found an increase in AFC 3 months postoperatively, but both authors, like Celik et al., suggested that AFC is a less reliable ovarian reserve marker in the presence of an endometrioma [3, 44].

The use of AMH and AFC as ovarian reserve markers before and after surgical treatment of endometriomas continues to be debated. In a recent meta-analysis, Muzii et al. challenged the conclusion that AMH declines after removal of an endometrioma [48]. These investigators emphasized that the meta-analyses by Raffi et al. and Somiglinana et al. that showed a reduction in AMH after excisional surgery had extremely heterogeneous results which limits the impact of the findings [41, 42]. Muzii et al. suggested that, in fact, AFC may be the preferred ovarian reserve marker in this situation. Moreover, the data from their systematic review indicated that AFC is reduced both before and after surgery but that this incremental decrease due to surgery is not significant. They recommended that future research is needed to better clarify the role of AMH and AFC in assessing the impact of surgery on ovarian reserve.

There are several proposed mechanisms in which laparoscopic cystectomy may worsen ovarian reserve including accidental removal of healthy ovarian cortex, thermal damage from coagulation of bleeding vessels, and surgical-related local inflammation [44]. Several authors have shown histologic evidence of damage to the ovarian cortex after laparoscopic cystectomy and that healthy ovarian tissue with primordial follicles is often inadvertently removed during cystectomy, particularly if the tissue is approaching the hilus [49–51]. Matsuzaki et al. demonstrated that ovarian tissue found on endometrioma cyst wall specimens was 10 times more frequent than on other benign cyst wall specimens after using the laparoscopic stripping method [22]. Transvaginal ultrasound of the postoperative ovary after endometrioma cystectomy has also shown a significant decrease in residual ovarian volume which possibly leads to diminished ovarian reserve [52]. Risk factors for removing more ovarian parenchyma during a cystectomy include cyst size and preoperative medical treatment [22, 49]. Although it was a small retrospective study, Roman et al. found an increase in the volume of ovarian tissue removed with an increase in cyst diameter [49]. In contrast, in their prospective study of 77 women with endometriomas, Romualdi et al. observed that more follicles were lost with surgery in women with smaller cysts. This correlation was only found in younger patients. The authors suggest younger women with small endometriomas should be warned that healthy ovarian tissue may be removed during surgery. Moreover, they observed that endometriomas that had a fibroblastic capsule were associated with an increased loss of follicular tissue after surgery compared to endometriomas with a fibrocystic capsule. The fibroblastic capsule was associated with more inflammation and also seemed to be less defined with respect to healthy ovarian cortex, thereby making it difficult to find a proper cleavage plane at the time of surgery [53].

As demonstrated by the preponderance of the clinical research literature, surgical removal of ovarian endometrioma cysts carries a significant risk of reduced fertility. Additionally, the best treatment to improve fertility in these affected women with or without surgical treatment is still a matter of debate. Many of these women will seek fertility treatment. Alborzi et al. conducted a randomized controlled trial to compare the ovarian response to controlled ovarian hyperstimulation (COH) and intrauterine insemination (IUI) between normal ovaries and ovaries previously treated by either laparoscopic ovarian fenestration and coagulation or ovarian cystectomy in 65 women. They also compared follicular response in 16 women with bilateral endometriomas in whom cystectomy was performed in one ovary and fenestration and coagulation on the contralateral side. These authors concluded that there is no significant difference between excisional surgery and ablative surgery regarding ovarian response or pregnancy rates to COH/IUI. They, however, recommended that more studies with a larger number of patients are required to verify this conclusion [54]. To date, there have not been any further randomized controlled studies specifically examining the best surgical approach to endometriomas before COH/IUI or even expectant management versus surgical management before COH/IUI. In a retrospective cohort study of 96 patients who underwent operative laparoscopy to treat endometriosis-related infertility, Gandhi et al. found that women with stage III/IV endometriosis should be offered in vitro fertilization (IVF) therapy if they have not achieved pregnancy spontaneously within the first few months after surgery rather than COH/IUI. They did not specify whether any of these patients had endometriomas [55].

Similarly, there are no randomized controlled trials that compare nonintervention for ovarian endometriomas before IVF versus surgical resection before IVF. Garcia-Velasco et al. did, however, conduct a case-control study in which 56 patients with endometriomas proceeded directly to IVF treatment and 133 patients first underwent laparoscopic cystectomy before IVF. They found no difference in the number of mature oocytes retrieved, fertilization rate, implantation rate, or pregnancy rate between these two groups of women. They concluded that although surgical excision before commencing IVF does not compromise fertility, it does not offer improvement either [56].

Some studies have found decreased responsiveness and a decreased number of oocytes retrieved in women with endometriomas, while others have not [57]. Nevertheless, the majority of studies have not detected a difference in fertility outcomes. Suzuki et al. found that the number of retrieved oocytes as well as number of embryos transferred was reduced in women with endometriomas versus those with tubal factor. Yet there was no difference in fertilization rate, implantation rate, pregnancy rate, or live birth rate [58]. On the other hand, in a retrospective case-control study of 81 women with unilateral endometriomas, the AFC and number of retrieved oocytes did not differ between the affected and contralateral healthy ovary, regardless of cyst size, during an IVF cycle [59]. Similarly, in a prospective cohort study of 29 women with unoperated unilateral endometriomas undergoing IVF, Filippi et al. reported no difference in number of oocytes retrieved or fertilization rates in the affected versus unaffected ovary [60]. Benaglia et al. reported that even the presence of unoperated bilateral endometriomas does not affect the quality of the oocytes retrieved or chances of pregnancy [61]. In an earlier study, Reinblatt et al. demonstrated that the quality of embryos obtained from IVF is not reduced in women with bilateral endometriomas [62].

Multiple studies have indicated that surgical excision of these cysts does not improve pregnancy rates before IVF. Some have suggested that surgery may have a negative impact on IVF parameters [63]. In a meta-analysis by Tsoumpou et al., there was no improvement in IVF outcome after laparoscopic cystectomy. This study found no significant difference in the ampules of gonadotropins required, oocytes retrieved, embryos available for transfer, or clinical pregnancy rates between expectant management and surgical intervention [64]. In a prospective study comparing operated and unaffected ovaries in women who previously underwent unilateral excision of endometriomas, Ragni et al. did find a lower number of developing oocytes and retrieved oocytes from the operated ovary. However, there was no difference in fertilization rates or high quality embryos in these women [65]. These results are in accordance with those of Bongioanni et al. who found reduced AFC in women with prior cystectomy compared to women with tubal factor infertility but no difference in IVF pregnancy rates per cycle [66]. This again indicates that surgical excision of endometriomas does not confer any additional benefits prior to IVF. Cyst size has been implicated in a reduction in ovarian response during IVF treatment. Somigliana et al. reported that there is a 53% reduction of follicles >15 mm at time of hCG administration in ovaries with prior endometrioma excision versus contralateral intact ovaries. This reduction was observed regardless of cyst diameter (≤3 cm versus >3 cm) [67]. On the other hand, Tang et al. observed that damage to ovaries is more severe in terms of AFC, number of dominant follicles, and number of oocytes retrieved during an IVF cycle if an endometrioma ≥4 cm is removed [68].

Finally, several authors have argued that IVF, rather than surgery, may be a more successful option in women with recurrent endometriomas who have had previous surgical excision [69, 70]. In a retrospective study of 173 patients, the recurrence rate and re-recurrence of endometriomas after laparoscopic cystectomy were reported to be as high as 45.1% and 45.5%, respectively. Women who achieved postoperative pregnancy were found to have less chances of recurrence [71]. As stated above, surgical excision procedures may lead to ovarian damage in women with endometriosis and the additive effect of multiple surgeries may be even more detrimental to a woman's subsequent fertility.

### 3.3. Surgical Technique

Surgical technique in treating endometriomas has been a point of controversy; however laparoscopic excision by stripping technique is one of the most widely used approaches [72]. A recent meta-analysis showed that stripping technique is a better method than drainage or ablative surgery in terms of recurrence of pain symptoms, increasing spontaneous pregnancy rates, and decreasing recurrence and reoperation rates [17, 73, 74]. However, the authors concluded that there is insufficient evidence to recommend excisional surgery over ablative surgery with respect to pregnancy outcome after COH and IUI (1.40, 95% CI 0.47 to 4.15) [17]. The recommendations from the recent ESHRE guidelines for women with endometriomas undergoing surgery for infertility or pain are to perform laparoscopic excision rather than drainage and electrocoagulation of the endometrioma wall [37].

Opponents of cystectomy cite that the biggest drawback is removal of healthy ovarian cortex which leads to decrease in ovarian reserve. Some evidence has indicated that cyst drainage and vaporization or thermal coagulation may be less harmful to ovarian reserve. Tsolakidas et al. compared the laparoscopic stripping technique to a three-step approach (laparoscopic drainage, GnRH analogue for 3 months, and laparoscopic CO_2_ laser vaporization). These authors documented that AMH does not decline in women who underwent the ablation procedure compared to those who underwent the stripping procedure [43]. Var et al. randomized 48 patients with bilateral endometriomas to either laparoscopic ovarian cystectomy or coagulation. After the intervention the ovarian volume and AFC were reduced in both groups, but the reduced AFC after cystectomy was statistically significant. The cystectomy group also had significantly reduced ovarian response to ovulation induction [75].

In an effort to combine the benefits of both the stripping procedure and ablation procedure, Donnez et al. proposed a technique consisting of excising a large part of the endometrioma wall using the stripping technique and then using CO_2_ laser on the remaining endometrioma wall when approaching the hilus. Six months after the surgery, the ovarian volume and AFC in the operated and contralateral unaffected ovary were not significantly different. The spontaneous pregnancy rate was 41% after a mean follow-up of 8.3 months and only one case (2%) of recurrence was noted [76]. The authors did not report IVF rates and therefore more research is needed to assess the benefits of this combined technique on infertile women who do not achieve spontaneous pregnancy following surgery.

## 4. Conclusion

Ovarian endometriomas, which are a common feature of endometriosis, create a complex situation for infertile patients. Both the presence of endometriomas and surgical excision of endometriomas appear to be damaging to ovarian function and ovarian reserve. The mechanism by which these endometriotic cysts cause infertility may be related to mechanical stretching of the ovarian cortex as well as an inflammatory reaction with cytotoxic oxidative stress and increased fibrosis. Surgery is the predominant clinical practice for the treatment of endometriomas and the most common surgical technique is stripping of the endometrioma. Although this technique has several advantages including increasing spontaneous pregnancy rates, it has also been shown to further reduce ovarian reserve. Nevertheless, the presence of an endometrioma does not appear to adversely affect IVF outcomes and surgical excision of an endometrioma does not appear to improve IVF outcomes. The most recent evidence suggests that asymptomatic infertile patients, especially those that are older, have diminished ovarian reserve, have bilateral endometriomas, or have had prior surgical treatment, would benefit from proceeding directly to IVF. This treatment path would avoid the risks associated with surgery and reduce the time to achieve pregnancy for the patient. In patients who have symptoms, intact ovarian reserve, unilateral cysts, or sonographic features concerning for malignancy or who are not planning on pursuing IVF, surgery may well be indicated. These women need to be adequately counseled on the potential for decrease in ovarian reserve.

There is a lack of randomized controlled studies comparing nonintervention to surgical excision of an endometrioma before IVF in infertile women. Future research is needed to better identify surgical techniques, such as aspiration with sclerotherapy and drainage with endometrial ablation using plasma laser energy, which may cause less ovarian damage. Since many women may now decide on expectant management of their endometriomas, further study regarding the implications of this treatment path are necessary. For example, studies that evaluate whether the ovarian damage mediated by an endometrioma is acute or progressive over time, as well as further investigation of the cytotoxic impact of an endometrioma on surrounding follicles during an IVF cycle will be especially informative.
